# Brain activity during shadowing of audiovisual cocktail party speech, contributions of auditory–motor integration and selective attention

**DOI:** 10.1038/s41598-022-22041-2

**Published:** 2022-11-05

**Authors:** Patrik Wikman, Artturi Ylinen, Miika Leminen, Kimmo Alho

**Affiliations:** 1 Department of Psychology and Logopedics, University of Helsinki, Haartmaninkatu 3, PO Box 21, 00014 Helsinki, Finland; 2 Department of Neuroscience, Georgetown University, Washington, DC USA; 3 Analytics and Data Services, HUS Helsinki University Hospital, Helsinki, Finland; 4 Advanced Magnetic Imaging Centre, Aalto NeuroImaging, Aalto University, Espoo, Finland

**Keywords:** Cortex, Attention, Motor cortex, Sensorimotor processing, Language

## Abstract

Selective listening to cocktail-party speech involves a network of auditory and inferior frontal cortical regions. However, cognitive and motor cortical regions are differentially activated depending on whether the task emphasizes semantic or phonological aspects of speech. Here we tested whether processing of cocktail-party speech differs when participants perform a shadowing (immediate speech repetition) task compared to an attentive listening task in the presence of irrelevant speech. Participants viewed audiovisual dialogues with concurrent distracting speech during functional imaging. Participants either attentively listened to the dialogue, overtly repeated (i.e., shadowed) attended speech, or performed visual or speech motor control tasks where they did not attend to speech and responses were not related to the speech input. Dialogues were presented with good or poor auditory and visual quality. As a novel result, we show that attentive processing of speech activated the same network of sensory and frontal regions during listening and shadowing. However, in the superior temporal gyrus (STG), peak activations during shadowing were posterior to those during listening, suggesting that an anterior–posterior distinction is present for motor vs. perceptual processing of speech already at the level of the auditory cortex. We also found that activations along the dorsal auditory processing stream were specifically associated with the shadowing task. These activations are likely to be due to complex interactions between perceptual, attention dependent speech processing and motor speech generation that matches the heard speech. Our results suggest that interactions between perceptual and motor processing of speech relies on a distributed network of temporal and motor regions rather than any specific anatomical landmark as suggested by some previous studies.

## Introduction

In the classical Wernicke–Lichtheim–Geschwind model of speech processing, it was postulated that the neural mechanisms supporting *speech perception are* largely separate from the mechanisms supporting *speech production*^1^*.* However, several later studies have shown that this division is not absolute, as speech production systems are recruited even when perceiving speech^2^ and speech production is strongly dependent on speech perception^3–8^. Such interactions between perceptual and motor systems fall under the umbrella term of sensorimotor integration (auditory–motor integration when referring to such effects in the auditory modality). There are currently two highly influential functional models of the brain processes involved in sensorimotor integration in the auditory system. In the dual stream model of Rauschecker and Scott^9^ (see also^10,11^), connections between the posterior auditory cortex, the temporo-parietal cortex, and ultimately motor and prefrontal cortical areas constitute the dorsal stream. This stream supports mapping between auditory and motor sound representations to enable sound production, correction of production errors, and auditory localization. Connections between the anterior portions of the auditory cortex, the inferior frontal cortex and the dorsolateral prefrontal cortex, in turn, constitute the ventral stream involved in auditory object processing, identification and finally processing of sound related semantics and meaning^12^. In the dual stream model developed by Hickok and Poeppel^13^ (see also^3,14^), there is less emphasis on the sensory processing in the auditory cortex, and more emphasis on speech semantic and syntactical processing in the ventral stream. In addition, their model emphasizes the importance of processes in the left posterior planum temporale (left pPT), which they refer to as the sylvian–parietal–temporal area (Spt). This area is considered important for translation between auditory and motor systems, that is, translation of auditory information into motor commands, and vice versa. The view that the left pPT is related to auditory–motor translation is based on results showing similar enhanced activation in the left pPT during listening to speech and during covert speech production^4,15–17^. Further, the model suggests that processes in the left pPT region is particularly important for actions that are novel and nonautomatic such as repetition of vocalizations made by other individuals^3,18^.

According to the aforementioned dual stream models^3,11^, sounds (including speech) are either processed in order to distinguish their identity and meaning in the ventral stream, or in order to couple them with motor representations (auditory–motor integration) in the dorsal stream. These models are, however, largely based on results from studies utilizing perceptual tasks only. One way of testing this prediction is designing tasks that either (a) demand processing of the meaning of the sounds or (b) demand processing of the sound information in order to couple it with sound production. Speech repetition tasks have been studied as an example of the latter case, because repeating speech involves converting perceived sounds into a motor plan for speech production^19–25^. Simple speech production, in turn, is based on more direct activation of motor programs and thus demands less sensorimotor control^3,18,21,25^. Accordingly, our previous study^25^ found that repeating vowel sounds activated regions in dorsal stream regions such as the left posterior planum temporale (pPT) and inferior parietal lobule (IPL) more strongly than producing self-selected vowels. However, this repetition-related activation did not depend on the difficulty of the speech repetition task, as has been suggested in Hickok’s model^3^. That is, repeating native language phonemes caused similar activations as repeating non-native (novel) phonemes, even though the repetition of phonemic vowels should benefit from the well-learned representations of one’s native language^26^. Similarly, in a previous study by Parker Jones et al.^21^, no differences in activation patterns were found between repetition of real words compared to repetition of pseudowords in posterior regions of the auditory cortex, even though one could argue that pseudoword repetition would demand more auditory–motor translation, as one cannot rely on stored word forms. Together, these results cast doubt on the assumption that posterior auditory regions such as the left pPT are particularly important for auditory–motor actions that are novel and nonautomatic. However, in both these previous studies, the participants repeated short speech segments (phonemes or words). Thus, it might be that longer (i.e., sentence level) repetition is needed to strongly engage auditory–motor translation systems in the left pPT during novel or nonautomatic speech repetition^25^. Recent studies (see, e.g., Ref.^27^) have also indicated that effects in the auditory cortex are stronger when using complex lifelike speech stimuli consisting of full sentences, rather than simple phonemes^25^ or words^21^, because the variability in such stimuli is larger causing less neural adaptation^28^. Also, lifelike speech tasks may engage participants more than less naturalistic tasks^27^. To our knowledge, however, it has not been tested whether manipulating the difficulty of a speech repetition task with lifelike sentence-level stimuli, for example, by modulating the sound quality of the ‘to-be-repeated’ speech modulates activations in the left pPT.

A similar but slightly different task than overt repetition that has been used to study auditory motor interactions is the so-called speech shadowing task^5^ (also called fast repetition task), where participants immediately repeat (‘shadow’) longer segments of heard speech. This task has been argued to circumnavigate explicit phonological or semantic processing of the heard speech^29^. Thus, shadowing should involve direct transformation of information between the auditory cortex and the parts of motor cortex that control the articulatory system.

Speech shadowing also strongly engages selective attention to the repeated sounds. In fact, the shadowing technique was originally developed to engage attention to one of two dichotically presented speech streams^30^. Despite the use of the shadowing task in early studies on attention to speech^30^, to our knowledge, there is no previous work specifically studying the neural processes related to attentive processing of speech with distracting speech sounds (i.e., cocktail-party speech) using the shadowing technique. In contrast, previous studies on the neural basis of cocktail-party speech processing have generally used listening tasks, where participants attentively listen to one speech stream, while ignoring another speech stream, and answer questions related to the topics of the attended speech stream. Previous studies using these kinds of paradigms have shown that selective attention to cocktail-party-like speech, strongly modulates core regions in the ventral stream, such as the superior temporal gyrus and sulcus (STG and STS, respectively), as well as the inferior frontal gyrus (IFG), all of which are associated with processing of perceptual speech properties^27,31–34^. This type of attentional modulation has been shown to be independent of spatial and feature based attention, operating directly on auditory object representations^35^ and probably therefore modulating information processing in the ventral stream^36^. Because previous studies on attentional processes during cocktail-party speech have in general only used listening tasks, it is difficult to evaluate whether the neural networks implied are specific for listening tasks or generalizable to other types of auditory tasks (such as the aforementioned shadowing task). A recent study from our lab^37^ suggests that there is some level of task generality in neural networks involved in selective attention to cocktail-party speech. That study found attention-related modulations in the auditory cortex and inferior frontal cortex to be similar when people attentively listen to one of two presented speech streams as when they perform a phonological task on the attended speech stream. However, that study also demonstrated differences between the tasks, because several regions in the somatosensory and motor cortex were more engaged when listeners process the phonological aspects of the speech than when they focus on the semantic content of the speech. Furthermore, several brain regions that have previously been implicated in semantic and social processing^38^, such as the temporal pole, angular gyrus and medial frontal regions were more strongly activated during the active listening task (where participants paid attention to the semantics of the speech) than the phonological task. In that study, we suggested that this dissociation was due the motor speech system being more engaged when participants attended to the phonological aspects of speech than when they merely tried to understand the meaning of speech (in accordance with previous results that have shown that the speech motor system is involved in perceptual processes related to speech phonology^39^). Thus, it would be important to study whether brain activations differ when participants listen to cocktail-party speech in order to understand its meaning compared to repeating the designated speech steam (shadowing) and therefore paying attention to the production related aspects of the heard speech.

In the current functional magnetic resonance imaging (fMRI) study, participants were presented with audiovisual (AV) dialogues between two people, with concurrent task-irrelevant speech present in the background. The AV dialogues had either good or poor auditory quality and either good or poor visual quality, the poor auditory or visual quality being expected to reduce speech intelligibility (e.g., Refs.^40,41^). The participants performed four different tasks: (1) a shadowing task, where they overtly repeated the speech in the AV dialogue as fast as possible (while ignoring the irrelevant speech stream); (2) a listening task, where they listened to the AV dialogue (while ignoring the irrelevant speech stream) and answered questions about its semantic content afterwards; (3) a motor control task where they ignored the speech streams altogether and overtly counted numbers in an ascending order; (4) a visual control task where they ignored the speech streams altogether, focused on a fixation cross below the AV speaker’s face, and counted the times the cross rotated. The focus of this study was to compare activations during the shadowing task, which was expected to modulate activations in the dorsal processing stream, to the listening task, which was expected to modulate activations in the ventral processing stream. However, since both the shadowing task and the listening task demanded attention to the relevant speech sounds, we included visual and motor control tasks to determine the effect of attention to the speech stimuli. In the resulting factorial design, the shadowing task demanded selective attention to AV speech and motor speech production; the listening task demanded selective attention to AV speech and processing of the meaning of the speech stimuli but no motor speech production; the motor control task demanded no attention to perceived speech but did demand speech production; and the visual task demanded neither attention to speech nor speech production. Thus, we aimed to reveal neural mechanisms related to selective attention to speech at the presence of irrelevant speech (the shadowing and listening tasks vs. the motor and visual control tasks); production of speech sounds (the shadowing and motor control tasks vs. the listening and visual control tasks); and most importantly effects that cannot be solely attributed to the perception or the production of speech (i.e., auditory–motor interaction effects), when the attended speech was repeated aloud during the shadowing task (where converting perceived sounds into heard speech is necessary).

We hypothesized that effects related to attention to cocktail-party speech would be found along the STG and STS, as well as in the IFG^27^, irrespective of the task (shadowing or speech listening). Speech production during both the shadowing and motor control task would be associated with effects in the motor cortex (the primary, premotor, and supplementary motor cortex), as well as in the sensory regions of operculum and insula^42^. Auditory–motor interaction effects related to speech shadowing, in turn, were expected in the posterior auditory cortex (including the left pPT), parietal cortex, and premotor cortex^10^. Further, in accordance with Rauschecker’s dual stream model^9^, we hypothesized that listening to speech with the intention to repeat it (the shadowing task) would engage posterior regions of the auditory cortex, while listening to speech with the intention to understand its meaning (the listening task) would engage anterior regions of the auditory cortex. In addition, the tasks were factorially combined with good or poor auditory and visual speech qualities of the AV dialogues. Based on conclusions made from our previous study^25^ we hypothesized that when the intelligibility of the speech is poor, the participants would need to more strongly engage direct auditory–motor translation processes during the speech shadowing task, which would cause stronger activations in the left pPT.

## Materials and methods

### Participants and ethics statement

Nineteen participants took part in the present study, but one of them was excluded from all analyses due to a technical error in data collection and another due to incorrect performance in the shadowing task. Thus, we analyzed data from 17 participants (mean age 25.6 years, range 19–39 years; 9 females). All participants were healthy native Finnish speakers with self-reported normal hearing (note, however, that no audiometry was conducted) and normal or corrected-to-normal vision, and they had no self-reported psychiatric or neurological disorders. All were right-handed as verified by the Edinburgh Handedness Inventory^43^. The participants gave a written informed consent and were monetarily compensated for their time (€15/h). The present experimental protocol was approved by the University of Helsinki Ethics Review Board in the Humanities and Social and Behavioural Sciences, and the study was conducted in accordance with the Declaration of Helsinki. Written informed consent was obtained from the individual(s) for the publication of any potentially identifiable images or data included in this manuscript (see also Refs.^26,31^).

### AV cocktail-party speech

AV dialogues between a female and a male speaker were used as stimuli. The dialogues covered emotionally neutral everyday topics (e.g., hobbies or weather; for details of the recording, editing and linguistic content of the videos, see^33^). Each dialogue had seven lines spoken alternately by the two speakers. The gender of the first speaker alternated from one video to another. The dialogue lines were 5.4 s long on average (range 4.9–6.1 s) and were always followed by a pause (mean pause duration 3.4 s, range 2.9–3.9 s). This resulted in ca. one minute per dialogue (range 55–65 s) in total.

A second speech stream (auditory only; passages from an audiobook; *The Autumn of the Middle Ages*, by Johan Huizinga, originally published in 1919) was added to each video. The audiobook, which is distributed freely by the Finnish Broadcasting Company (Yleisradio, YLE; https://areena.yle.fi/1-3529001), was spoken by a female native Finnish speaking actor. The pitch of the voice of the audiobook’s speaker was decreased to a mean of 0.16 kHz in order for it to be clearly distinguishable from the speakers of the dialogues, and it was low-pass filtered with a cut-off of 5 kHz (for details, see^33^). The volume of the audiobook was 3 dB lower than the volume of the AV dialogues.

**Figure 1:**
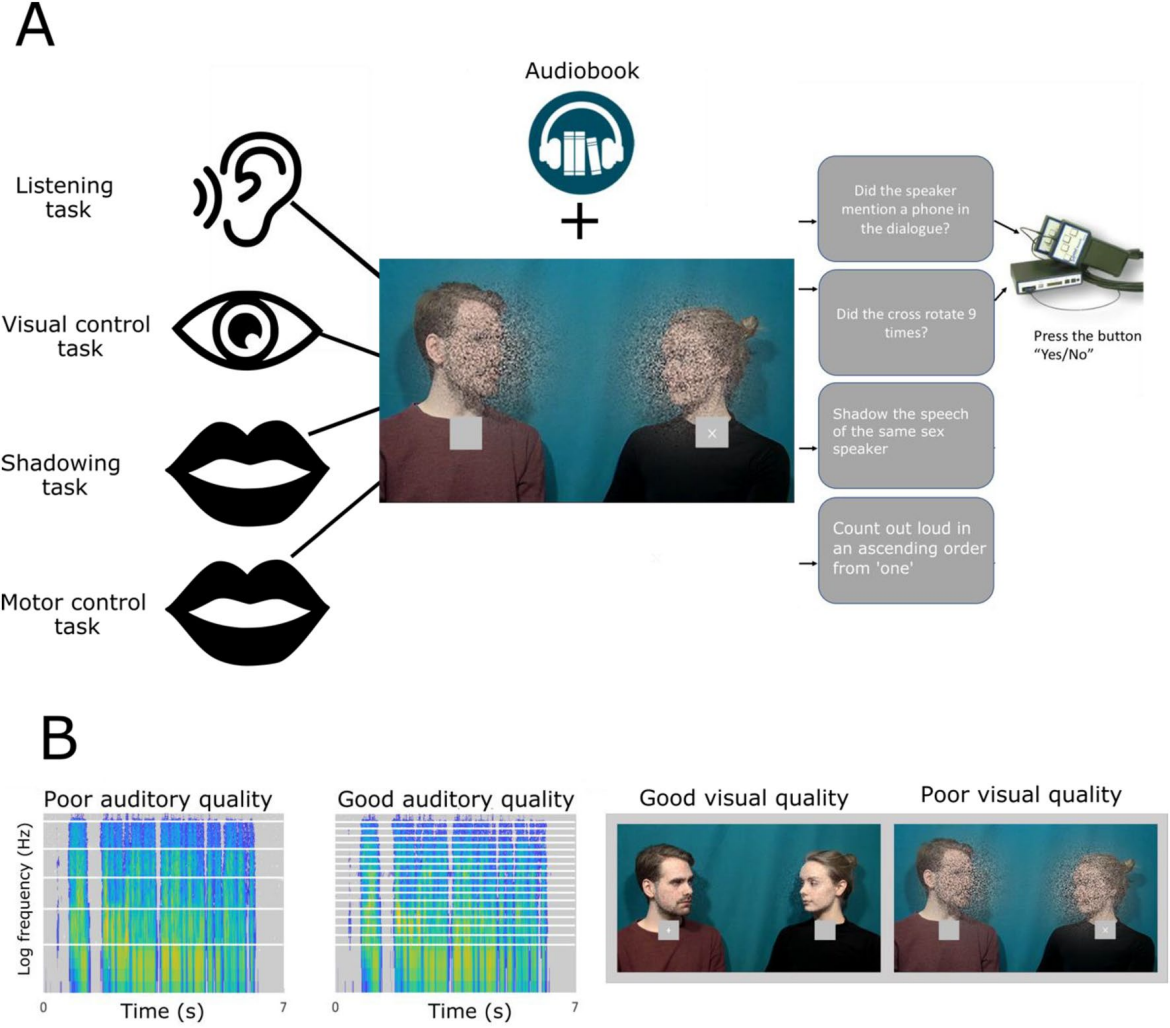
The audiovisual (AV) cocktail party design used in the current study. (**A**) The participants were presented with video clips (ca.1 min in duration) of a male speaker and a female speaker discussing neutral topics, such as the weather, while a continuous audiobook was played in the background. Speech from the two talkers alternated with a short break between talkers. The participants performed four tasks: (1) a listening task, where they attended to the dialogue while ignoring the audiobook and answered questions about each line of the dialogue immediately after the video-clip finished, and (2) a visual control task, where the participants ignored the dialogue and audiobook, and instead counted rotations of a cross presented below the neck of the talker who was speaking at the moment, (3) a shadowing task, where participant shadowed, that is, overtly repeated as quickly as possible the line of the speaker of the same gender as the participant themselves (i.e., male participants shadowed the male speaker’s speech and female participants shadowed the female speaker’s speech), (4) a motor control task where participants overtly counted from ‘one’ forward during the lines spoken by the speaker of the same gender as themselves. (**B**) Videos were presented at two levels of auditory quality: Poor auditory quality, where the audio stream of the dialogue was noise-vocoded^45^ with four logarithmically equidistant frequency bands above 0.3 kHz (i.e., the fundamental frequency was untouched), and good auditory quality, where it was noise-vocoded using 16 bands above 0.3 kHz (white horizontal lines on the spectrograms denote the frequency band borders). (**B**) Visual quality of the faces was modulated by masking the speakers’ faces with different amounts of dynamic white noise.

Noise-vocoding^44^ was used to manipulate the intelligibility of the attended speech streams. In this method, the amplitude envelopes of a speech stream in logarithmically divided frequency bands are used to modulate white noise. The intelligibility of noise-vocoded speech is dependent on the number of frequency bands used^34^. In the present study, in order to retain information on the gender of the speakers, the fundamental frequencies (F0; < 0.3 kHz) of the speech streams were left intact, and the frequencies from 0.3 to 5 kHz were noise-vocoded on two levels, that is, four bands vs. 16 bands (Fig. 1B), using Praat software (version 6.0.27^45^; https://www.fon.hum.uva.nl/praat/). The bandwidth borders for F0+ 4 bands were 0.300, 0.684, 1.385, 2.665, and 5.000 kHz, and the bandwidth borders for F0+ 16 bands were 0.300, 0.376, 0.463, 0.565, 0.684, 0.822, 0.982, 1.168, 1.385, 1.637, 1.929, 2.269, 2.665, 3.124, 3.658, 4.279, and 5.000 kHz (for further details, see^33^). Thus, we produced two auditory qualities, one with poor intelligibility and the other with intelligibility not differing from that of clear speech, as verified in a separate behavioural pilot experiment (n = 5^33^).

The visual quality of the videos was also varied on two levels. This was done by masking the speakers’ faces with visual noise (for details, see^33^). In the poor visual quality conditions, the faces were almost completely occluded by the noise, whereas in the good visual quality conditions there was very little noise (Fig. 1B).

For the visual control task (see “Tasks” section), a light grey box containing a white fixation cross was added below the face of both speakers in all videos (see Fig. 1A). At the beginning of each dialogue video a cross was present on the screen below the face of the speaker who would utter the first line. 1500 ms after the start of the first line, a second cross faded in below the face of the other speaker (fading in of the crosses rather than their abrupt appearance was used to avoid exogenous orienting to the crosses; cf.^46^). 500 ms after the end of each line, the fixation cross below whoever had been speaking faded out. The cross that faded out then faded back in 1500 ms after the next line started. This pattern was repeated until the end of the last line. Thus, most of the time there were two fixation crosses present on the screen. The cross rotated from ‘+’ to ‘×’ and vice versa, and the participants’ task was to detect these rotations (see “Tasks” section for details). There were always between 1 and 15 rotations of the cross per dialogue, with a mean of 7.2 rotations. The exact timing of each rotation was randomly distributed throughout the videos, but there was always at least 1.25 s between each rotation.

During one of the motor control tasks, the order of the spoken lines in the dialogues was shuffled, resulting in dialogues with no coherent semantic context. We used shuffled videos in this task as there were a limited number of original videos^33^. Shuffled videos were also used during a training session, as the participants practiced performing the tasks of the present study (see “Procedure” section). The shuffled videos were originally created for another study^27^, by cutting lines from various dialogues and combining them into a new video. The transitions between the cuts were edited so that they were scarcely noticeable (for details, and examples see supplementary video materials in Ref.^27^). The shuffled videos were used during the training in order to avoid showing the same normal dialogue videos in the training and in the actual experiment.

### Tasks

In the experimental session, the participants performed five different tasks. The tasks that the participants performed were: (1) a listening task), (2) phonological task (not reported here), (3) visual control task, (4) shadowing task, and (5) motor control task. Effects related to three of the tasks (the listening, visual and phonological tasks) are reported elsewhere^37^. In the present study, we include the listening and visual tasks only to disentangle their relation to the shadowing and motor tasks, which have not been reported previously.

In the shadowing task, the participants immediately repeated the speech of the speaker that was of the same gender as the participant themselves (i.e., males shadowed the male speaker, and the females shadowed the female speaker). In the motor control tasks, the participants counted aloud numbers ascendingly starting from ‘one’ whenever the same-gender speaker was speaking. The participants performed these two tasks only during the speech of the speaker that was of the same gender as the participant, because people are better at shadowing speech with a similar fundamental frequency to their own fundamental frequency in spontaneous speech^5^, and because overt speech production causes instantaneous motor artefacts in the blood oxygen level dependent (BOLD) signal measured with fMRI. However, the brain activity related to speech production causes a delayed BOLD signal (peaking 5 s after the start of the motor production of speech). Thus, by having the participants to shadow ca. 5 s of speech and then stay silent for ca. 10 s, effects related to speech production were expected to peak when there were no motor speech artefacts because the participant was not overtly speaking at this point (for a description of how this method decreases motor artifacts in studies utilizing overt speech, see^47^). During both tasks, the participants were instructed to only passively listen to the lines spoken by the opposite-gender AV speaker.

In the listening task (reported also in Ref.^37)^, the participants were instructed to watch and listen to the dialogue and keep their gaze on whoever was speaking. After the presentation of each dialogue, the participants answered seven yes–no statements regarding the content of the dialogue. The statements concerned the content of each of the seven lines in the dialogue (e.g., “One of the speakers had attended a concert”) and were presented in the order of the lines. Half of the questions were correct, and half were false.

In the phonological task (reported in Ref.^37^, but not here), the participants were instructed to listen to the two speakers and search for occurrences of the phoneme [*r*] in the speech stream, the number of which they reported after the dialogue.

In the visual control task (reported also in Ref.^37^, the participants were instructed to focus on the fixation cross that was below the face of whichever speaker was speaking and ignore the dialogue. The task was to count how many times the crosses rotated from ‘+’ to ‘×’ and vice versa. After each dialogue video, the number of rotations was reported (i.e., statements of form “There were *x* rotations of the cross” with *x* being “1–2”, “3–4”, “5–6”, “7–8”, “9–10”, “11–12”, and “13–15”). The cross that was below the face of the speaker not speaking at a particular time was not to be attended, and never rotated. Further, the fading out of the cross that was fixated on, as a line ended, acted as a cue for the participants to shift their gaze to the second cross.

The participants were instructed to ignore the audiobook during all tasks. The fixation crosses were present in all conditions, but the participants were instructed to ignore the crosses in all but the visual control task.

The conditions in the experiment constitute a 2 × 2 × 2 × 2 repeated measures factorial design, with the factors Motor (shadowing and motor control vs. listening and visual control), Auditory attention (shadowing and listening vs. motor control and visual control), Auditory quality (poor vs. good) and Visual quality (poor vs. good).

The shadowing and motor control tasks are considered as motor (M_1_) tasks because both demand motor speech production. The listening and visual control tasks are considered as non-motor (M_0_) tasks because the participants did not produce speech during these tasks. The shadowing and listening tasks are considered auditory attention (A_1_) tasks, because both demanded attention to the AV speech. The motor control and visual control tasks are considered no auditory attention (A_0_) tasks, as no attention to the speech was required. Thus, the shadowing task (M_1_, A_1_) demands motor speech production and attention to the speech input; the motor control task (M_1_, A_0_) demands motor control of speech but no attention to the AV speech stimuli; the listening task (M_0_, A_1_) demands no motor speech production but attention to the AV speech and processing of speech semantics; the visual control task (M_0_, A_0_) demands neither motor speech production nor attention to the AV speech stimuli.

This design was planned to distinguish effects related to: (1) general speech motor production (motor main effect) and hearing one’s own speech (i.e., an area is activated equally by both the shadowing and motor control task vs. the listening and visual control task); (2) attention to AV speech (attention to speech main effect, i.e., an area is activated by both the shadowing and the listening task vs. the motor control task and visual control task); (3) auditory–motor interaction (the use of this term in the manuscript will refer generally to any effect in the neural data that cannot be assigned solely to the perception of the speech or the overt production of speech), which is expected to occur specifically during the shadowing task when the heard speech is relevant for the produced speech output, but not during the other tasks that only demand attentive processing of speech or mere motor production of learned speech patterns; (4) activations that are related to the processing of the speech semantics, which would be present specifically during the listening task, but not the other tasks. As the focus of the present study is on motor and task-related effects, we only examined main effects and interactions that contained either the Motor speech production or the Attention to speech factor.

### Procedure

One or two days before the fMRI session, all participants completed a training session of approximately 1 h. The purpose of this session was to maximize the participants’ behavioural performance in the scanner by familiarizing with the tasks. In the training session, the participants were first given instructions about the stimuli and the tasks, after which they practiced performing the tasks on a laptop. A set of six videos not used in the actual experiment were used as training stimuli. Of the videos used in the training session, five videos were shuffled (see “AV cocktail party speech” section, Ref.^27^).

In the whole experiment there were two runs, both including 20 dialogue blocks. The blocks consisted of one block of each of the five tasks in each of four audiovisual (AV) quality combinations. The order of tasks and specific dialogue videos within each run was randomised. For all tasks except the motor control task, the dialogue videos were selected from 36 dialogues originally created for a previous experiment (see^33^) that were randomly paired with the tasks. The same dialogue video was never presented more than once within a run.

Instructions on which task the participants were to perform next appeared on the screen before the presentation of each dialogue. A quiz followed immediately after each video. This quiz consisted of seven yes–no statements shown for 2 s each, to which the participants were instructed to answer by pressing a button with their right index finger for “yes” and with their right middle finger for “no”. After the statements, participants received feedback on their performance (i.e., how many questions out of 7 they answered correctly). The quizzes shown after the shadowing and motor control tasks consisted of questions that were unrelated to the semantic content of the dialogues (e.g., ‘Finland is located in Asia’ and ‘Penguins can swim’). The stimulus videos were presented on light grey background. The length of a block was always 85 s, which comprised (1) instructions (2 s), (2) a grey screen with a fixation cross indicating which speaker would speak first (2–12 s), (3) the dialogue video (55–65 s), (4) a quiz (14 s), (5) feedback (2 s). The audiobook clips started 500–2000 ms before the onset of the videos and stopped at video offset. A rest block of 40 s occurred between the 10th and 11th blocks. During the rest block, the participants were to look at a small fixation cross in the middle of the screen.

The experiment was controlled using Presentation 20.0 (Neurobehavioral Systems, Berkeley, CA, USA). Videos were presented with a mirror mounted on the head coil. The videos were presented at an approximate size of 26° horizontally and 15° vertically in visual angles, from a viewing distance of ca. 38 cm. Sounds were delivered binaurally through earphones including canal tips that also acted as earplugs (Sensimetrics Model S14; Sensimetrics, Malden, MA, USA). The intensity of the sounds was determined individually to be pleasant but loud, and it was ~ 80 dB SPL at the tip of the earphone. Scanner noise (approximately 102 dB SPL, as measured in the head coil) was also attenuated with viscoelastic mattresses around and under the head of the participant and inside the coil. Verbal responses during the shadowing and motor control tasks were recorded with a noise-cancelling MRI safe microphone (FOMRI II, Optoacoustics Ltd., Or-Yehuda, Israel) that was attached to the head coil and reached in front of the mouth of the participant.

### (f)MRI data acquisition

fMRI data was acquired using a 3 Tesla Magnetom Skyra whole body scanner (Siemens Healthcare, Erlangen, Germany) with a 20-channel head coil. Two functional runs of 703 volumes were acquired per participant. For the first two participants, from whom the runs consisted of 714 volumes, the excessive 11 volumes at the end of a run were deleted. The fMRI data comprised 43 oblique axial slices of T2*-weighted echo planar images (EPI; TR 2600 ms, TE 30 ms, flip angle 75°, field of view 192 mm, slice thickness 3.0 mm., 64 × 64 voxel matrix; in-plane resolution 3 mm isotropic). A high-resolution anatomical image was obtained (MPRAGE sequence, 176 × 256 × 256 voxel matrix, in-plane resolution 1 mm isotropic), after the fMRI runs. Simultaneous electroencephalography (EEG) was recorded from all participants during the fMRI session with a 32-channel MR compatible EEG cap (Braincap MR 32-ch, Easycap, Herrsching, Germany) and an MR compatible amplifier (BrainAmp MR plus, Brain Products GmbH, Gilching, Germany). Unfortunately, these data had to be left unanalyzed, as after data collection we noticed that there was a jitter of tens of milliseconds in the fMRI pulse timings, rendering it impossible to remove MRI artifacts from the EEG data. Note that simultaneous measurement of fMRI and EEG with a low-density EEG cap (as in the present study) has little or no effects on the signal-to-noise ratio of fMRI data in field strengths of 3 Tesla^48^.

### Behavioral data analysis

The percentage of correct answers per dialogue was used as a measure of task performance in the listening task. In the visual tasks, the distance of the participant’s answer from the correct answer was used instead. For example, if a dialogue contained 7 or 8 rotations, and the participant answered that there were 5 or 6 occurrences, the distance from the correct answer was 1. This measure was used because it reflects performance more accurately than the simple number of correct and incorrect answers: detecting 7 out of 10 task-relevant events is better than detecting only 5, which is reflected in the distance to the correct answer but not in the simple number of correct answers to the yes/no questions. Note, however, that in case of the distance measure, chance level performance cannot be assessed, as it varies depending on the number of task-relevant occurrences per video. Missing responses were considered as errors in all tasks. For none of the participants were the responses missing altogether in the visual task and the distance could always be defined. Behavioral performance in the four tasks was analyzed using separate repeated-measures ANOVAs conducted with IBM SPSS Statistics 27 (IBM SPSS, Armonk, NY, USA). In each ANOVA, the two factors were Auditory Quality (poor, good) and Visual Quality (poor, good). The results were visualized using custom-made Python scripts.

### Analysis of the shadowing responses

The vocal responses were transcribed and rated by two experienced research assistants that were unfamiliar with the goal of the current study using the software program ELan^49^. The transcribers were asked to count the number of correct words per line, and separately report each stuttering, phonetic and semantic substitution error.

Next, we calculated response times for each repeated word by calculating the lag from the onset of the word in the video stimuli to the onset of the vocal response. The onset of the overt vocal repetition for each shadowed word, mean correct shadowing responses/line and response time/word were used in the fMRI analysis (see “(f)MRI data pre-processing and analyses” section).

For the counting task, transcribers were asked to determine the number of numbers produced per line of a dialogue and the onset and duration of the vocal responses.

### (f)MRI data pre-processing and analyses

Pre-processing and first-level analyses of the fMRI data were performed using FEAT (FMRI Expert Analysis Tool) Version 6.00, part of FSL (FMRIB’s Software Library, http://www.fmrib.ox.ac.uk/fsl). Registration of fMRI volumes to the high-resolution structural image of the participant was carried out using FLIRT^50,51^, and pre-processing included motion correction using MCFLIRT^51^, slice-timing correction, non-brain removal with BET^52^, and high-pass temporal filtering (with a cut-off of 130 Hz). For all further fMRI analyses, the data were then projected to the Freesurfer^53^ average surface space (fsaverage) using the Freesurfer function mri_vol2surf.

In total three first-level analyses were preformed, where a general linear model (GLM) was fit to the time series data of each voxel in each run. In the first GLM, the main effects and interaction terms of a 2 × 2 × 2 × 2 ANOVA were built into the first level model, with factors Motor speech production (shadowing and motor control vs. listening and visual), Attention to AV speech (shadowing and listening vs. visual and motor control), Auditory quality (good, poor) and Visual quality (good, poor). For the shadowing and motor control tasks, the model contained a regressor for each line uttered by the participant, defined as the time interval during which the participant shadowed a particular line or counted numbers out loud. One such regressor was created separately for each auditory and visual quality combination, modelling all the lines uttered in that condition. The participants were to shadow/count during either three or four lines per dialogue, depending on the gender of the first speaker, as they always shadowed the speech of the same-gender speaker, and there were always seven spoken lines per dialogue. To keep the number of utterances that were modelled constant, we included only the first three shadowed/counted lines for each AV quality condition in the ANOVA. The fourth line possibly uttered by the same-gender speaker, as well as the lines spoken by the different-gender speaker during which the participant did not shadow speech or count numbers, were modelled with one separate regressor of no interest. For the listening and visual tasks, in turn, the regressors corresponded to the spoken lines in the dialogue. To match the number of events modelled in these tasks with those modelled in the shadowing and motor control tasks per block, we included into the ANOVA only the first three lines spoken by the speaker of the same gender as the participant. The rest of the lines were modelled in the regressor of no interest.

The second and the third GLMs were constructed to examine activations during the shadowing task in more detail. In the second GLM, two regressor were included that modelled each line during which the participant shadowed speech or overtly counted numbers, respectively for the shadowing and motor control tasks. For the shadowing task, the regressor value for each shadowed line depicted the percentage of correctly shadowed words for that line. Correspondingly for the motor control task, the regressor value corresponded to the number of overtly counted words per line divided by the maximum number of counted words per line in that block. The values of these regressors were demeaned by taking the average value per run and subtracting it from all the values of that run. Separate regressors were also included for each AV quality condition in the shadowing and motor control tasks in order to account for AV quality related effects. All other tasks as well as instructions and quizzes were modelled in a separate regressor of no interest.

In the third GLM, we aimed to examine brain activations that were either positively or negatively associated with the participants shadowing response times (RTs). That is, a positive association means that the brain regions activity rises with longer RTs and a negative association that the brain activity increases with shorter RTs. The RTs corresponded to the time lag between the onset of a word in the dialogue and the onset of the corresponding shadowed word. In this GLM we included a regressor to model each correctly shadowed word with values corresponding to the RT of that word. The RT values were demeaned by taking the average RT value per run and subtracting it from all RT values. To account for AV quality related effects, regressors for each of the four AV quality conditions of the shadowing task were included in this model as well, while all other tasks, instructions, and quizzes were modelled with one regressor of no interest. We will refer to this analysis as intra-individual correlation with the shadowing RTs (in the “Results” section). The six basic motion parameters were also included in all GLMs as confounds.

Group-level analyses were performed using Freesurfer version 6.0.0 and a one-sample t-test performed with the mri_glmfit function. Clusters were defined using permutation inference^54^ with the initial cluster forming threshold z set at 3.1. Only familywise error (FWER p < 0.05) corrected clusters (corrected across different whole brain maps) are reported.

### Peak activity analysis

For each participant the MNI-peak coordinate on the of the freesurfer projected data along the STG or STS was determined for the contrasts: listening task and shadowing task vs. baseline; listening task vs. visual control task, and shadowing task vs. motor control task. A straight line was defined visually in both hemispheres running across the STG (see Fig. 3), each coordinate was thereafter projected into this line using custom python scripts implementing linear vector multiplication, in order to determine its relative anterior–posterior position in relation to the STG.

### Left posterior planum temporale (pPT) region-of-interest analysis

The mean % signal change was calculated for each combination of the shadowing or the motor control task and the four auditory and visual quality levels in the left pPT. The left pPT region of interest (ROI) was based on the motor main effect cluster occurring in this region (see Fig. 5).

## Results

### Behavioral performance

**Figure 2:**
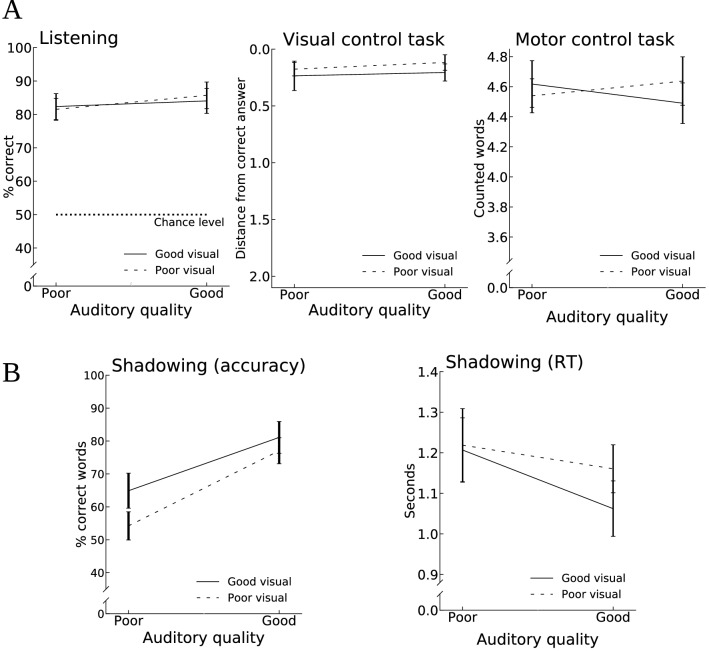
Behavioral performance in the four tasks and four audiovisual (AV) quality conditions (± SEM). (**A**) In the listening task, performance was above chance level in all conditions, but AV qualities had no significant effects on performance. In the visual control task, audiovisual qualities had no effect on task performance. In the motor control task, we only report the amount of overtly uttered numbers. (**B**) In the shadowing task Auditory quality had a significant effect on both accuracy and response time, and Visual quality had a significant main effect on accuracy.

To study task performance in conditions with varying speech intelligibility (i.e., conditions with different auditory and visual qualities), separate 2 × 2 repeated-measures ANOVAs with factors Auditory quality and Visual quality were conducted for each of the four tasks (see Fig. 2). In the listening, visual control, and the motor control task (Fig. 2A), no significant main effects or interactions were found (listening: F_1,16_ < 1.23, p > 0.28 for all effects; visual: F_1,16_ < 1.1, p > 0.33 for all effects; motor control: F_1,16_ < 1.94, p > 0.18 for all effects. Two 2 × 2 repeated-measures ANOVAs were conducted for the shadowing task (Fig. 2B). Regarding the percentage of words correctly shadowed per line, both Auditory quality and Visual quality had a significant main effect on performance (Auditory quality: F_1,16_ = 43.9, p < 0.001, ηp^2^ = 0.73; Visual quality: F_1,16_ = 5.7, p = 0.03, ηp^2^ = 0.26), whereas the interaction of these two factors was not significant (F_1,16_ = 1.1, p = 0.3). Regarding the response time of shadowed words, the main effect of Auditory quality was significant (F_1,16_ = 7.8, p = 0.013, ηp^2^ = 0.33), the main effect of Visual quality approached significance (F_1, 16_ = 4.2, p = 0.057, ηp^2^ = 0.21), but the interaction of these two factor was not significant (F_1,16_ = 2.2, p = 0.15).

We also analyzed separately the phonemic and semantic shadowing errors in the four AV qualities of the shadowing task using two-way repeated measures ANOVAs with the factors Auditory quality (poor, good) and Visual quality (poor, good). Only one significant effect of Auditory quality was found for the semantic errors (F_1,16_ = 4.6, p < 0.05), all other effects were non-significant. As can be seen in Table 1, the overall error-rate was very low for all error types and therefore brain activity related to the errors was not analyzed (e.g., some participants had no errors in their shadowing responses).

**Table 1:** Percentage of different error types out of all shadowed words by audiovisual (AV) quality (± SEM).

Auditory quality	Poor	Good	Poor	Good
Visual quality	Poor	Poor	Good	Good
Phonological error	0.8 (0.4)	1.1 (0.4)	0.6 (0.4)	1.1 (0.4)
Semantic error	3.1 (0.6)	1.7 (0.4)	3.3 (1.1)	2.0 (0.5)
Stuttering error	0.7 (0.3)	0.7 (0.2)	0.6 (0.3)	1.3 (0.6)
Total	4.6	3.5	4.5	4.4

### fMRI results

**Figure 3:**
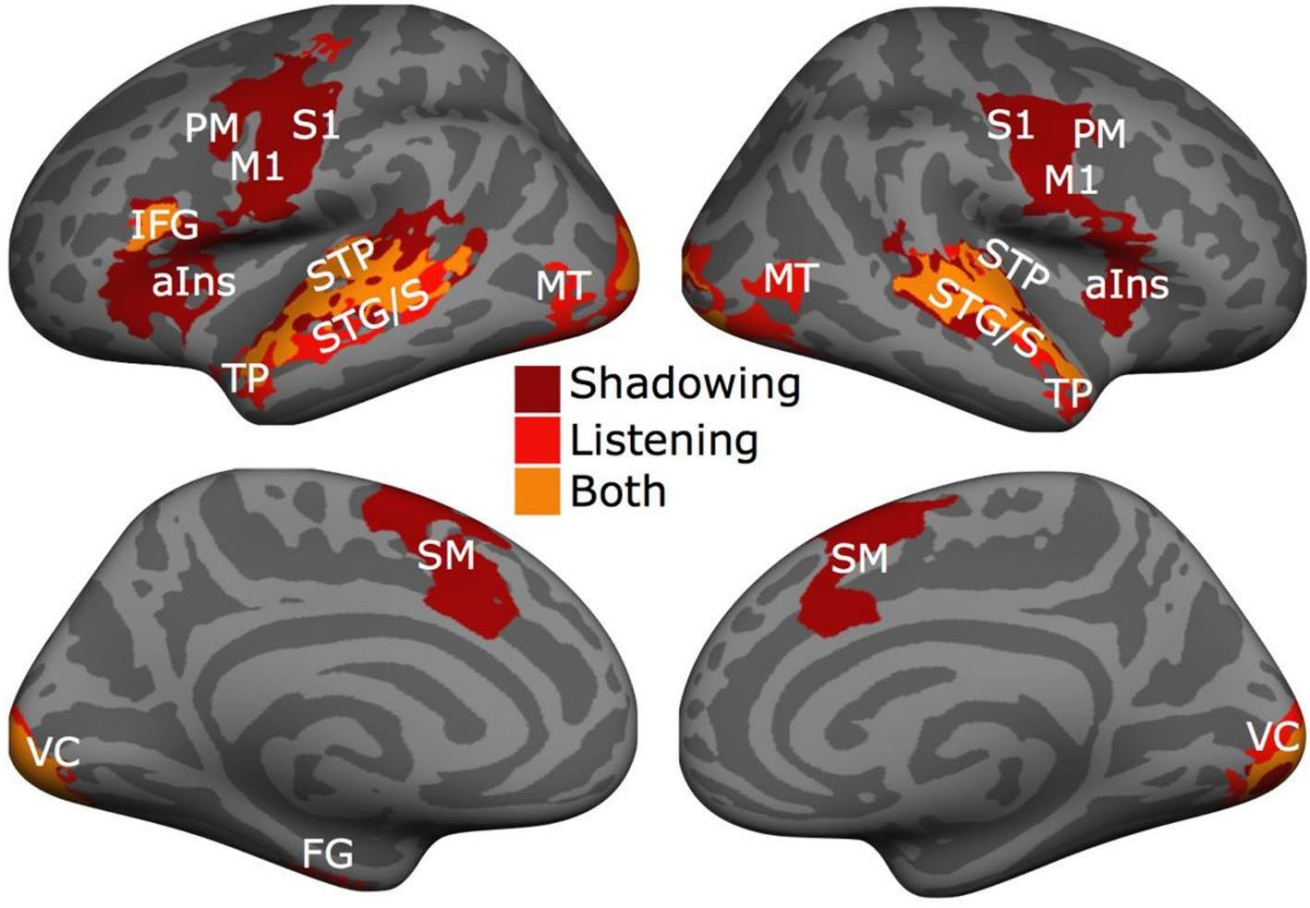
The listening task and the shadowing task activated partly overlapping regions in distributed cortical networks. Significant clusters (initial cluster threshold z = 3.1; permutated cluster significance p < 0.05, family-wise error rate, FWER corrected across all whole brain analyses) for the listening task vs. silent baseline (Bright red and orange), and the shadowing task vs. silent baseline (dark red and orange). Upper row: Lateral views of the inflated left and right hemisphere (lighter gray denotes gyri and darker gray sulci). Lower row: Medial views of the left and right hemisphere. *STP* supratemporal plane, *STG/S* superior temporal gyrus/sulcus, *TP* temporal pole, *MT* middle temporal visual area, *aIns* anterior insula, *IGG* inferior frontal gyrus, *M1* primary motor cortex, *PM* premotor cortex, *S1* primary somatosensory cortex, *VC* visual cortex, *FG* fusiform gyrus, *SM* supplementary motor cortex.

First, we determined the degree of overlap between activations during the AV speech listening task and the shadowing task (task vs. silent baseline between the task blocks). As seen in Fig. 3, activations overlapped bilaterally in the supratemporal plane, STG, STS, and visual cortex of each hemisphere, and in the left IFG. The listening task was associated with independent activations bilaterally in the anterior STS and temporal pole, middle temporal visual area (MT) and the fusiform gyrus (FG). In contrast, the shadowing task was associated with independent activations bilaterally in the posterior supratemporal plane, posterior STG/STS, anterior insula, and premotor cortex, as well as in the supplementary and primary motor cortex and primary somatosensory cortex.

**Figure 4:**
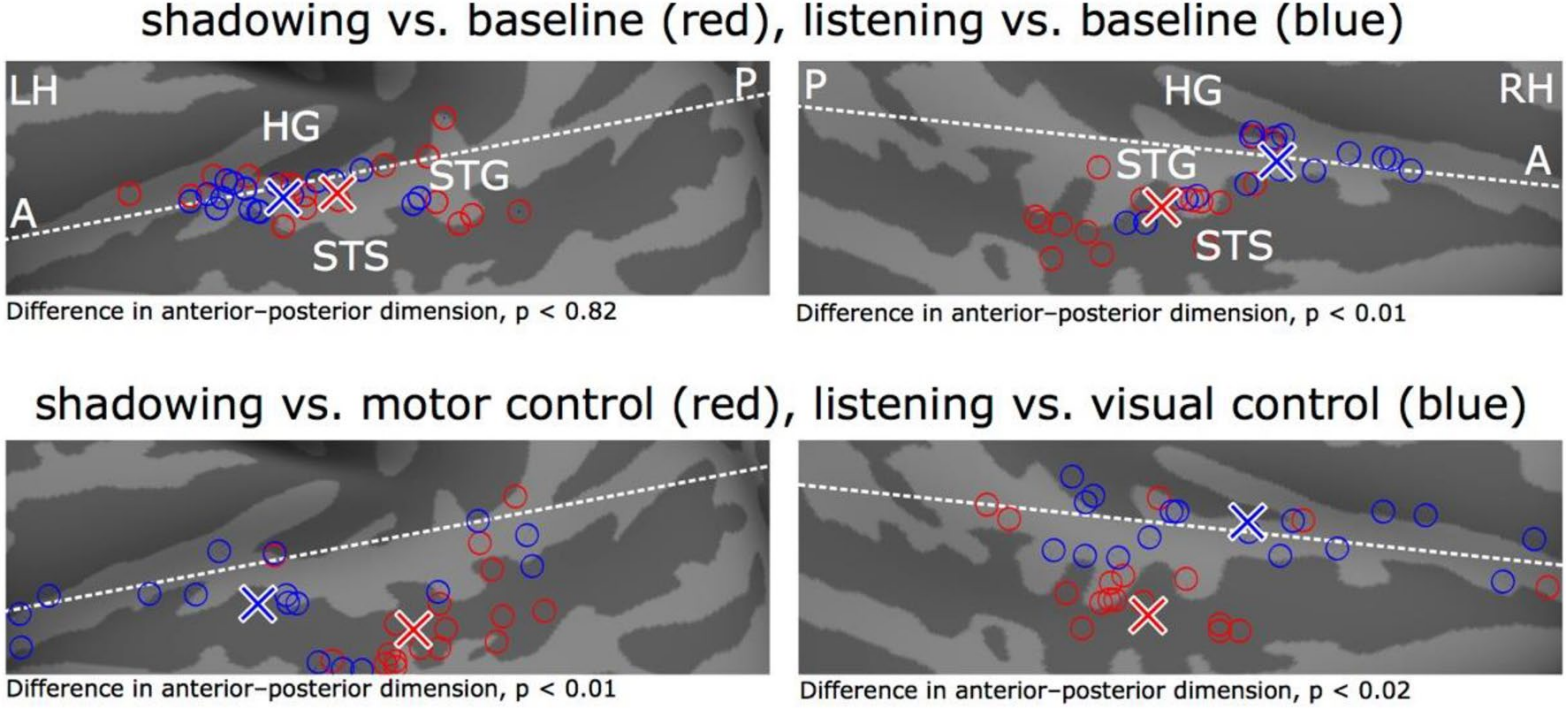
The peak coordinates for the AV speech listening task are significantly anterior to the peak coordinates for the shadowing task in the STG/STS. The white lines denote the anterior–posterior dimension in the STG, individual peak coordinates for the listening task (vs. baseline) are denoted by blue circles (mean blue cross) and the corresponding peak coordinates for the shadowing task are denoted by red circles (mean red cross). The upper row shows the peak coordinates for the task vs. baseline in the left and right STG/STS (lighter gray denotes gyri and darker gray sulci) The lower row shows the peak coordinates for the listening and shadowing tasks contrasted with their respective control task, that is, visual control and motor control task, respectively, controlling for stimulus and speech production related effects. *LH* left hemisphere, *RH* right hemisphere, *A* anterior, *p* posterior, *HG* Heschl’s gyrus, *STG* superior temporal gyrus, *STS* superior temporal sulcus.

As hypothesized, this analysis showed that the neural networks activated when listening to AV speech in the STG/STS were anterior to those associated with shadowing AV speech. However, this does not yet indicate that there is a significant difference in the STG/STS anterior–posterior activity distributions between these two tasks. Therefore, we calculated the peak coordinate in the STG/STS for each participant separately for the listening task (vs. the baseline; see “Peak activity analysis” section) and shadowing task (vs. the baseline). This analysis revealed that the mean peak activation during the AV speech listening task in the STG/STS was significantly anterior to the mean peak activation during the shadowing task in the right hemisphere (Fig. 4, upper row: t_15_ = 4.2, family-wise error rate, FWER corrected p < 0.02; note one participant was excluded in this analysis because they did not show any activation above baseline in the contrasts in the STG/STS). In the left hemisphere, there was no significant difference after FWER correction (t_16_ = 2.6, FWER corrected p < 0.08).

The significant difference in peak activations between the two tasks could, however, be related to the fact that the shadowing task demanded speech motor production and the participants heard their own voice during the overt shadowing responses. These factors have been shown to influence activations in the auditory cortex in a complex manner (see^25,55^). Therefore, we examined the peak activations for the contrasts: listening task vs. visual control task and shadowing task vs. motor control task. These contrasts should not be influenced by either the stimulus dependent processing of the AV stimuli, nor the factors relating to simple motor production of speech. Importantly, the peak coordinates for these contrasts did also show a clear anterior–posterior difference between the two tasks (Fig. 4, bottom row, t_16_ > 3.3, FWER corrected p < 0.02).

**Figure 5:**
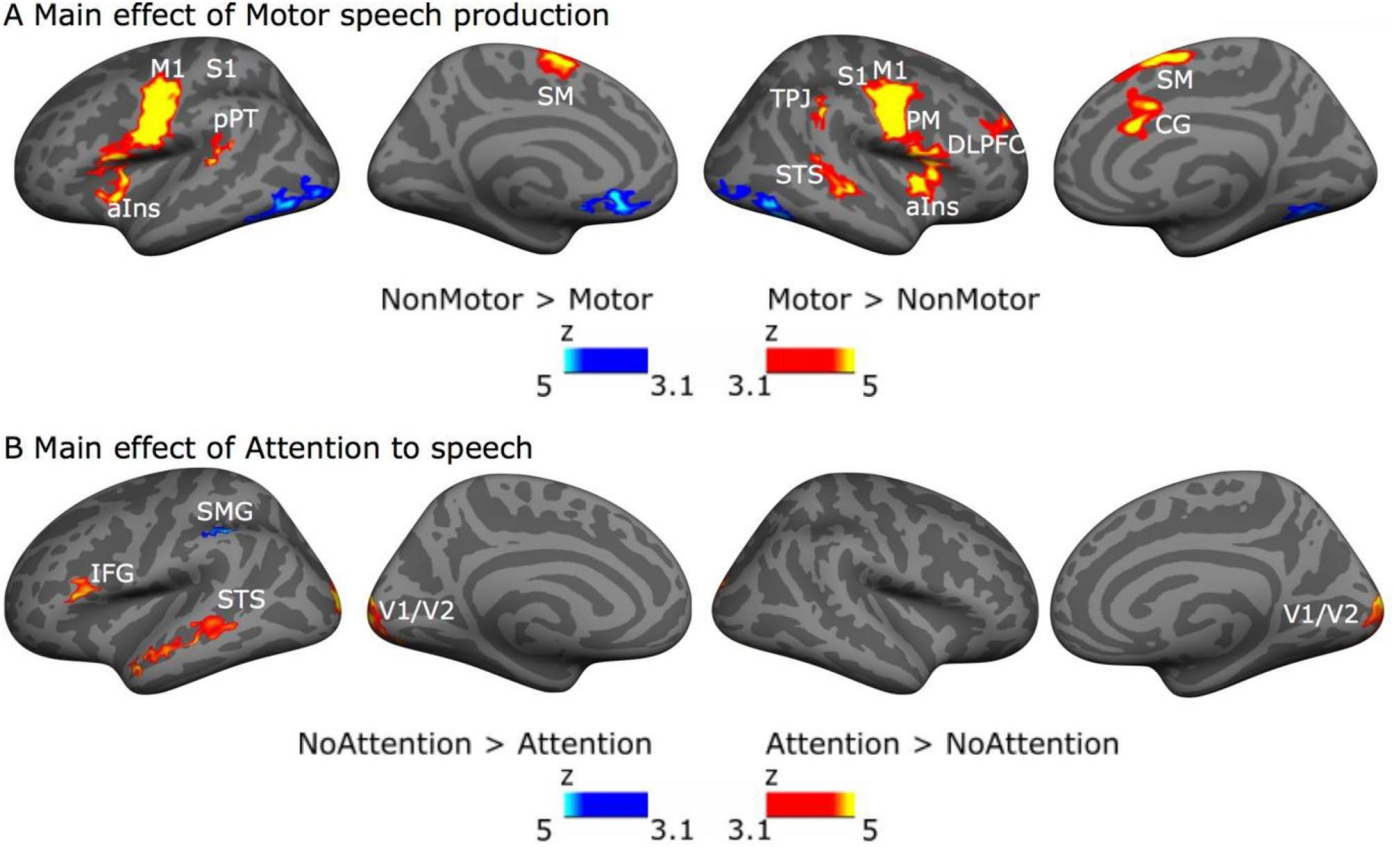
The omnibus ANOVA with factors Motor speech production, Attention to AV speech, Auditory quality and Visual quality revealed extensive main effects of Motor speech production and Attention to AV speech. (**A**) Significant clusters (initial cluster threshold z = 3.1; permutated cluster significance p < 0.05, FWER corrected) for the main effect of Motor speech production. Clusters where the motor tasks (shadowing and motor control task; Motor) activations were stronger than the non-motor tasks (AV speech listening and visual control task; NonMotor) are shown in red/yellow, the converse in blue/cyan. From left to right: lateral and medial views of the inflated left hemisphere and lateral and medial views of the right hemisphere (lighter gray denotes gyri and darker gray sulci). (**B**) Clusters where the tasks that demanded attention to AV speech (shadowing and AV speech listening task; Attention) activations were stronger than those during the tasks not demanding attention to AV speech tasks (motor control and visual control task; ignore) are shown in red/yellow, the converse in blue/cyan. *STS* superior temporal sulcus, *aIns* anterior insula, *IFG* inferior frontal gyrus, *M1* primary motor cortex, *PM* premotor cortex, *S1* primary somatosensory cortex, *V1/V2C* visual area ½, *SM* supplementary motor cortex, *TPJ* temporoparietal junction, *pPT* posterior planum temporale, *DLPFC* dorsolateral prefrontal cortex, *SMG* supramarginal gyrus.

Next, we performed a whole cortical surface omnibus 2 × 2 × 2 × 2 repeated-measures ANOVA with the factors Motor speech production (motor response vs. non-motor response), Attention to AV speech (attend vs. ignore), Auditory quality (good vs. poor), Visual quality (good vs. poor). The main effect for Motor speech production was significant bilaterally in the anterior insula, premotor, supplementary motor, primary motor, and primary somatosensory cortex, as well as in the right-hemisphere dorsolateral prefrontal cortex, right-hemisphere posterior STS, left-hemisphere pPT, and in the right-hemisphere temporo-parietal junction (Fig. 5A, red/yellow). These clusters were associated with significantly stronger activations during the motor tasks than the non-motor tasks. In contrast, the non-motor tasks activated bilaterally the fusiform gyrus and the orbitofrontal cortex more strongly than the motor tasks (Fig. 5A, blue/cyan).

The main effect of Attention to AV speech was significant in the visual cortex (V1, V2) bilaterally, and in the left IFG and the STG where the shadowing and listening tasks a were associated with stronger activity than the visual and motor control tasks (Fig. 5B, red/yellow). In contrast, the left supramarginal gyrus was activated more strongly during the visual and motor control tasks than during the listening and shadowing tasks (Fig. 5A, blue/cyan), possibly because the control tasks demanded counting (see^27^).

**Figure 6:**
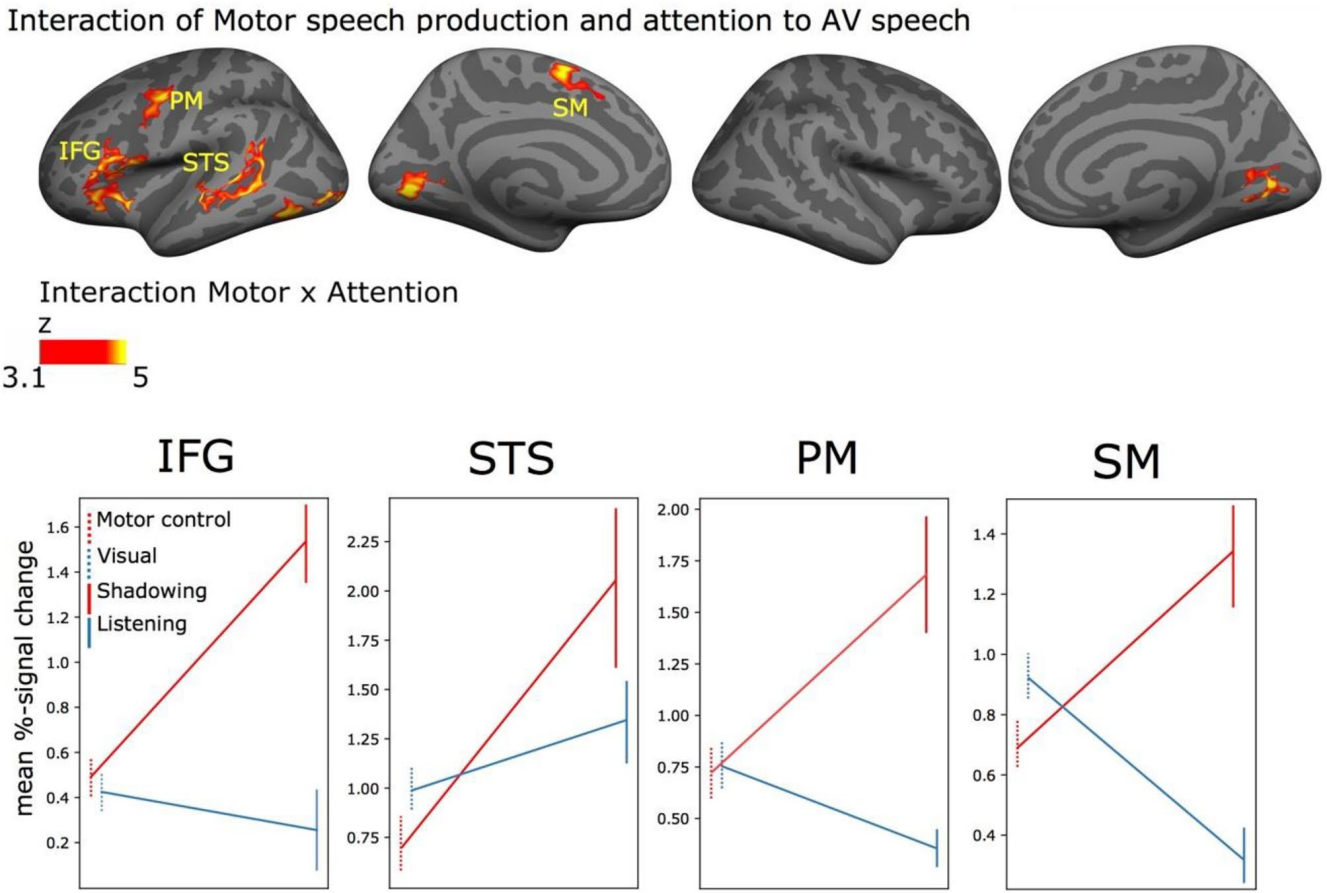
Significant interaction between Motor speech production and Attention to AV speech were found in left hemisphere auditory, motor and language regions. Top row: Significant clusters (initial cluster threshold z = 3.1; permutated cluster significance p < 0.05, FWER corrected) for the interaction Motor speech production × Attention to AV speech. From left to right: lateral and medial views of the inflated left hemisphere and lateral and medial views of the right hemisphere (lighter gray denotes gyri and darker gray sulci). Bottom row: The mean % signal change (vs. baseline) in each of the tasks are plotted separately for select significant clusters, Plots for all interaction clusters are shown in Supplementary Fig. 1. Error bars represent ± SEM. *IFG* inferior frontal gyrus, *STS* superior temporal gyrus, *PM* premotor cortex, *SM* supplementary motor cortex.

The omnibus ANOVA also revealed extensive significant interactions between Motor speech production and Attention to speech, as can be seen in Fig. 6 (uppermost row). Some of these clusters exhibited a pattern suggestive of involvement in auditory–motor interaction (see “Materials and methods” section for a definition of the use of the term in the current manuscript), such as: (1) the cluster extending from the left IFG into the anterior insula and (2) the cluster in the possible face-hand region in the left premotor cortex, (3) the STS and (4) supplementary motor cortex. In these regions, there was clear selectivity for the speech shadowing task, with little or no activity during the other three tasks (including the motor control task). Plots for all interaction clusters (including those in the occipital cortex are shown in Supplementary Fig. 1).

**Figure 7:**
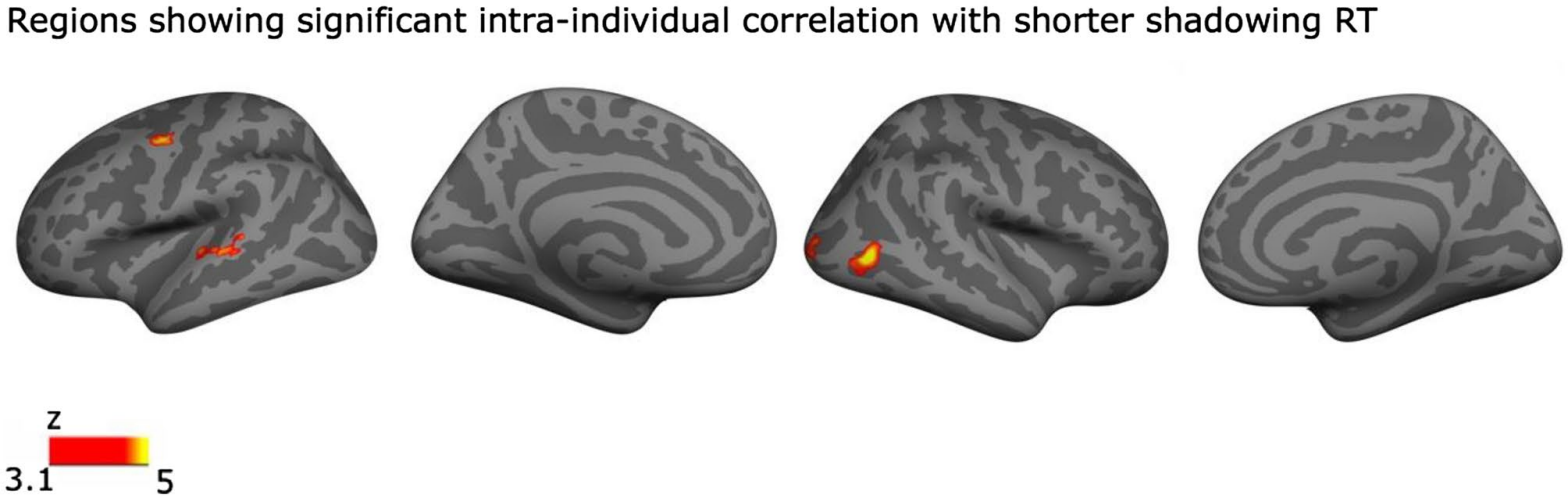
Significant clusters where shorter response times (RTs) were associated with stronger activations in the shadowing task. Significant clusters (initial cluster threshold z = 3.1; permutated cluster significance p < 0.05, FWER corrected) for the correlation effect (within subjects) between RT in the shadowing task and neural activations. From left to right: lateral and medial views of the inflated left hemisphere and lateral and medial views of the right hemisphere (lighter gray denotes gyri and darker gray sulci).

To see if activity in any region showed an association with performance in the shadowing task, we separately for each participants regressed their trial-to-trial performance (percentage correct, and response time) in the shadowing task using a whole brain GLM (see “(f)MRI data pre-processing and analyses” section). No region showed a significant association for the shadowing performance. However, significant correlations for response time (see “(f)MRI data pre-processing and analyses” section) were found in the left STG, left PM, right MT and right V1. In all these regions activations were stronger during faster shadowing responses (Fig. 7).

**Figure 8:**
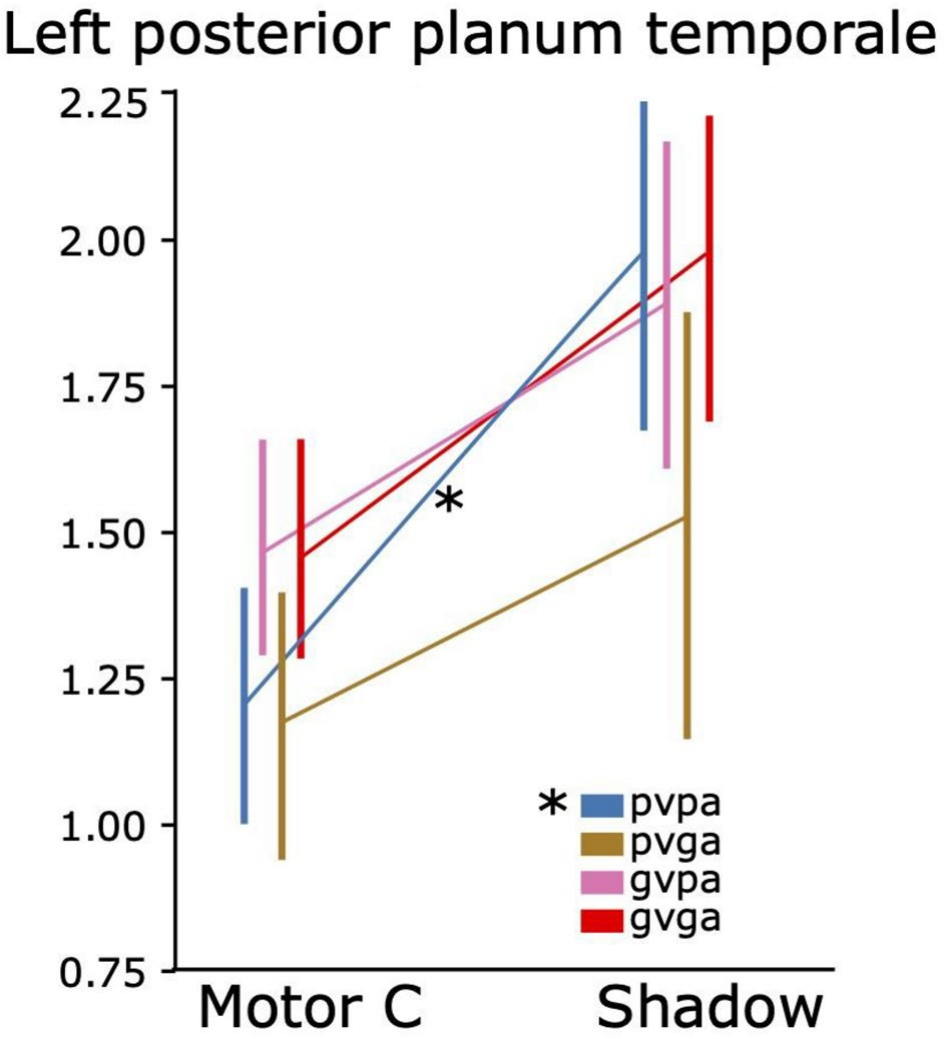
In the left posterior planum temporale (pPT), there was a significant difference in the mean % signal change between the shadowing (Shadow) condition with poor auditory and poor visual quality and the corresponding motor control (Motor C) condition. *p < 0.05, FWER corrected. *pv* poor visual quality, *pa* poor auditory quality, *gv* good visual quality, *ga* good auditory quality.

Lastly, in order to investigate our pivotal hypothesis that the left posterior planum temporale is involved in speech repetition especially when the quality of the speech input is poor (possibly involving more direct auditory–motor translation^3^) we first applied a repeated-measures ANOVA with the factors Task (Shadow vs. Motor control), Auditory quality (poor vs. good) and Visual quality (poor vs. good). This ANOVA revealed a significant Task main effect (F_1,16_ = 5.1, p < 0.04, ηp^2^ = 0.27), but no interactions. However, since we hypothesized that there would be a significant effect in the poor auditory and poor visual conditions, we conducted further pairwise t-tests (FWER-corrected) for each AV condition of the shadowing task, whether they differed from the respective motor control task condition. That is, if the region is specifically involved in auditory–motor translations there should be a difference between the two tasks when the AV quality is poor but not when it is good. The ROI for the left pPT was defined using the cluster depicted in Fig. 5. As seen in Fig. 8, there was a significant difference (t_16_ = 2.9, p = 0.04, FWER corrected, dʹ = 0.69) between the poor auditory and poor visual condition of the shadowing task and the corresponding motor control task, but not for any other pairwise condition.

## Discussion

The experimental design of the current study enabled us to study neural activations related to selectively attending to AV speech with the intention to understand the meaning of the speech (the listening task) or repeat it overtly (the shadowing task). This design also allowed us to study effects related to motor speech production and auditory–motor interactions during attentive speech processing. We replicated our previous findings that actively listening to AV speech activates broad regions of the STG/STS, the visual cortex, and IFG^27,33,37^. Importantly, in the current study, these effects were found irrespective of whether the participants listened to the speech in order to answer questions regarding its content (the listening task), or if they repeated the attended speech overtly (the shadowing task). Thus, this network appears to be fairly task invariant, as it is activated by attentive processing of AV speech both while attending to the semantic aspects of the speech input^27,33^, the phonological aspects of the speech input (see^37^, and when listening to it with the intention to overtly repeat it.

In our previous studies, selective attention to continuous AV speech (corresponding to the listening task of the current study) was associated with activation in the orbitofrontal cortex^27,33^. This region is classically implicated in processing of emotional and social information, moral judgments, and theory of mind^56–59^, but it has not been consistently activated in studies on speech processing^27,37,60^. Moreover, the stimuli of our studies were deliberately written to be as emotionally neutral as possible. In the current study the orbitofrontal activations were stronger during the listening and visual control tasks than during the shadowing and motor control tasks (see Fig. 5A). That is, one could argue that performing a motor task effectively diverts attention from the social aspects of the dialogue. Thus, the mere presentation of AV dialogues is insufficient to activate orbitofrontal regions (see also^37,61^), but the orbitofrontal cortex processes the social aspects of dialogues even when the dialogues are not particularly socially engaging, if the task does not demand attention to be diverted to other (e.g., phonological or production-related) aspects of the dialogues. We also expected to replicate the findings of our previous study^37^, where activations in the anterior temporal lobe, angular gyrus and medial frontal lobe were specifically activated during the listening task. Activations in these regions have strongly been linked to semantic processing of speech^38^. In the current study, however, we could not find any regions that showed significantly stronger activations during the listening task than the shadowing task (see Fig. 6, Supplementary Fig. 1). This possibly reflects that although processing of semantics was unnecessary during the shadowing task, and previous studies have suggested that speech shadowing circumnavigates explicit phonological or semantic processing of the heard speech^29^, participants covertly processed the meaning of the heard speech to some extent during shadowing. We suggest that in future studies one could use shadowing of coherent speech and incoherent (jabberwocky speech) to study the effects of covert semantic processing during speech shadowing.

Previous studies have found that processing sound identity and sound location activate overlapping areas in the auditory cortex^62–64^. However, there is evidence that the ‘center of gravity’ for processing of sound location is more posterior than the ‘center of gravity’ for processing of sound identity^62,65–67^. Here we show that a similar partial dissociation of activity to anterior vs. posterior aspects of the auditory cortex applies to the processing of speech sounds for understanding (listening task) vs. production (shadowing task). That is, activations during the shadowing task and the listening task overlapped to a large extent in the auditory cortex (see Fig. 3). However, peak activations in the STG/STS were significantly more posterior during the shadowing task than the listening task (Fig. 4 upper row). Meta-analyses (e.g., Ref.^68^) have indicated that processing of sound identity is associated with anterior portions of the auditory cortex, while processing of sound for production is associated with posterior portions of the auditory cortex. However, to our knowledge, previous studies have not tested this notion with the same stimulus materials and participants. The current study also indicates that attentional processing during the listening task vs. attentional processing during the shadowing task also shows a similar anterior–posterior distinction. That is, peak activations in the contrast listening vs. visual task, designed to capture attentional processing of speech sound while focusing on the identity aspects of the sounds (but controlling for stimulus related activity), were more anterior than the peak activations in the contrast shadowing vs. motor task, designed to capture attentional processing of sound while focusing on the production aspect of the sounds (but controlling for stimulus related effects and effects related to overt speech production). Here the activity peaks were found in the STS rather than the STG, which supports previous results showing that attention modulates processing in secondary auditory regions^66,69^. Similar findings regarding attentional processing of sound identity vs. sound location have also been reported^66^. Together, these results support the Rauschecker dual stream model^9–11,70^, where anterior parts of the STG/STS processes sound identity, whereas posterior parts process sound location and process sounds for production.

Different dual stream models^18,70,71^ postulate that there are neuronal mechanisms in the posterior auditory cortex that convey auditory information to motor brain regions during speech repetition and when correcting speech errors. In the current study, no activity in any brain region correlated with speech errors during the shadowing task, measured as percentage correct words repeated. This might have related to the fact that the shadowing responses were fairly accurate, possibly due to behavioral strategies adopted by the participants, i.e. choosing not to speak unless they could follow the dialogue, causing an overall small percentage semantic substitution or phonological errors compared to previous studies^5^. However, we found that shorter response times in the shadowing task were associated with stronger activations in the posterior STG immediately posterior to the HG. It is possible that this increased activation during fast repetition is indicative of auditory–motor interaction processes. That is, one could argue that during fast accurate responses participants directly translated the heard sounds into motor commands, involving specialized neuronal populations in the posterior auditory cortex, while when the responses were slow participants also relied upon alternative strategies involving identification of the words to be repeated. However, one could also maintain that the response latency effect in the posterior auditory cortex was related to stronger attentional engagement, that is, more strongly focused attention to the sounds during fast responses.

Two findings, however, speak against the latter interpretation: (1) Both in the current study and in the previous studies using the same AV speech paradigm^27,33,37^, the auditory quality of speech significantly modulated task performance and thus probably attentional engagement. However, auditory quality did not strongly modulate attention-related activations in the auditory cortex in the present study or previous studies. (2) In the current study, response latency did not only correlate with brain activity in the posterior auditory cortex, but also with brain activity in the dorsal premotor cortex. This region has not been associated with attentional engagement in previous studies using the current paradigm^27,33,37^, and thus is unlikely explained by stronger attentional engagement. On the other hand, the premotor cortex is an important hub in the dorsal stream of the dual stream model postulated by Rauschecker et al.^10,11^. In this model, the premotor cortex transforms desired behavior or heard sounds into appropriate motor commands, and a copy of the motor commands (corollary discharge or efference copy) is used to generate a forward model (transforms the motor command into predicted auditory consequences), which is then sent to auditory cortex. Within the auditory cortex the predicted sensation is compared to the actual incoming auditory information. If there is mismatch between the predicted and actual sensation, an error signal is generated and sent to premotor cortex, which initiates corrective action. Thus, the fact that in the present study both the premotor cortex and the auditory cortex are activated when participants have faster repetition responses could indicate activation of auditory–motor translation processes and processing of auditory or motor feedback in these regions.

There is converging evidence indicating that neuronal activity to self-produced sounds is suppressed in the auditory cortex. This seems to hold for both vocally produced sounds^72–74^ and manually produced sounds^25,55,75^. In the present study, there was no clear evidence for suppression of self-produced vocal sounds, as the listening and visual control tasks (no vocally produced sounds) did not activate the auditory cortex more strongly than the shadowing and motor control tasks (overt vocally produced sounds). However, it could be argued that hearing self-produced vocal sounds should cause stimulus dependent activation in the auditory cortex. Thus, if such activity is suppressed the net activation could be zero, leading to no difference between the vocal and the non-vocal tasks in the present study. Our recent study^25^, however, showed that the neuronal dynamics related to vocally produced sounds in the auditory cortex are fairly complex, consisting of both suppression and enhancement effects. Further in that study suppression effects related to vocal responding were smaller than during manual responding already before any overt utterance had been heard. Thus, smaller suppression effects during vocal responding could not be attributed to participants hearing their own utterances. The results from the current study corroborate this view by showing stronger activations during the shadowing task than the motor control task in the left posterior STG/STS. That is, these activations could be related to the fact that hearing and correcting one’s own vocalizations while repeating speech (shadowing task) is more important than when simply producing well learned words (motor control task) as suggested by our previous study^25^. Alternatively, one could argue that repeating heard sounds in the shadowing task would generate more speech production errors than producing numbers from memory (in the motor control task). Such mismatch between intended sounds and auditory feedback could generate error signals in the auditory cortex releasing auditory neurons from production related suppression^3,10^.

As expected, the current shadowing task and motor control task (Fig. 5A) activated a distributed network of motor, opercular, insular and inferior frontal regions that have previously been associated with control of speech production^42,76–79^. However, it is important to note that the inferior frontal regions were associated with a heterogenous set of effects in the current study. The pars opercularis region of the IFG showed a main effect of motor speech production (Fig. 5A), and an Attention × Motor interaction (Fig. 6) supporting the notion that the region is integral for control of speech production, as indicated by previous evidence suggesting its involvement in for example speech motor disorders such as apraxia of speech^80^. The pars-triangularis region of the IFG showed a main effect of Task (Fig. 5A), and a motor interaction (Fig. 6), suggesting this region might have more general speech processing functions, not restricted to speech production, per se. Additionally, the pars-orbitalis region of the IFG and regions of the IFS showed only an Attention × Motor interaction in the current study but no main effects, suggesting the area might be integral for auditory–motor processing, necessary during the shadowing task, but to a lesser degree during the other tasks (Fig. 6). However, previous studies from our lab have found that although the IFS is not activated strongly during speech listening tasks^27^, the region shows a decreasing temporal profile during listening tasks. Based on this we have suggested that the IFS is part of a ‘primary control network’ that orchestrate and facilitate processing in sensory regions, because such processes are required especially at the initial stages of listening to overlapping speech, before the task has become automatized. This was further supported by our previous finding that activations are stronger in the IFS when participants perform a novel phonological detection task compared to the same listening task of the current study^37^. That is, it could be argued that listening to speech is highly automatized and therefore mainly recruit sensory networks, while detecting phonemes in a speech stream is a much more novel task and therefore involves these higher-level control networks to a larger degree (see^81^). Therefore, it is important to consider that the stronger activations in the IFS during the shadowing task might not be related to auditory–motor interaction, per se. Rather activations in the IFS during the shadowing task could have been stronger than during the other three tasks used in the study because speech shadowing was a fairly or even entirely novel task for the participants, and thus demanded formation of new task plans and strategies (see^81^ for a review).

In Hickok and Poeppel’s model, the ‘Spt’ in the left posterior planum temporale acts as an interface between auditory and motor systems translating auditory sound information into motor commands and vice versa. Because the shadowing task demands online auditory–motor translation to a much stronger degree than the other tasks of the study, we expected to find an Attention × Motor interaction effect in the left pPT (which approximately overlaps with the ‘Spt’ region in the model postulated by Hickok et al.^15^). However, the whole brain analyses only revealed a motor main effect in the left pPT (Fig. 5) and no interaction effects. In contrast, evidence for auditory–motor interaction, which could include direct auditory–motor translation processes too, was found in multiple other regions, such as the posterior STG/STS, IFG, and premotor and supplementary motor cortex. These regions have consistently been associated with auditory–motor interactions during speech production^5,23,24,77,82^. However, as there are strong theoretical reasons to assume that the left pPT would be involved in auditory–motor translation processes we conducted an ROI analysis on the region specifically comparing mean signal change during the shadowing and motor control tasks. This analysis revealed that activations in the left pPT were significantly stronger during the shadowing task than during the motor control task. Further pairwise comparisons revealed that this main effect was driven by the difference between the conditions with poor auditory and visual quality. This result was expected, because one can argue that when the perceived speech is more difficult to comprehend, participants rely on direct translation of auditory information to motor output, while when the speech information is clear, participants can rely on alternative mechanisms to repeat the speech input. However, it is also important to note that the effect found in the left pPT was not strong (Cohens dʹ = 0.69) and similar trends as for the poor auditory and poor visual conditions can be seen for the other quality conditions. Thus, the left pPT might not be the most integral node for auditory–motor translation as suggested in Hickok and Poeppel’s model. Interestingly, there was a nearby cluster in the left posterior STG/STS that showed a significant Attention x Motor interaction (see Fig. 6). This might suggest that auditory–motor translation in the auditory cortex is distributed in posterior STP/STG/STS regions, rather than confined to the ‘Spt’.

## Conclusions

The present results corroborate our previous findings that attentive processing of AV speech in a cocktail-party-like setting is associated with modulations in a core network consisting of the STG/STS, the visual cortex and the IFG. The present results indicate that this occurs irrespective of whether listeners process speech in order to understand it (the listening task) or produce it (the shadowing task). However, these two tasks also show differential activation patterns, in accordance with the specific task demands (i.e., the listening task activates orbitofrontal regions associated with social cognition, while the shadowing task show strong involvement of speech motor regions). Furthermore, the ‘center of gravity’ of activations in the auditory cortex are more anterior when the listeners process the speech in order to understand it than when they process the speech in order to repeat it vocally. Similar findings have previously been reported for the processing of sound identity vs. sound location in the auditory cortex. Although, previous studies suggest that a dissociation into the respective auditory processing streams originates in the auditory cortex^12^, this notion has not been tested in the same study using exactly same stimulus materials during sound production tasks. Here we provide novel evidence for a partial dissociation in processing of sound object-properties in the anterior auditory cortex and sound production in the posterior auditory cortex*.* Thus, the current results strengthen the view that there are two parallel processing streams originating in the auditory cortex, where the anterior auditory cortex is involved in auditory object processing and identification, while the posterior auditory cortex is involved in auditory localization and mapping between auditory and motor speech representations to enable sound production. Additionally, effects that might arise due to auditory–motor interaction during the speech shadowing task were found in the IFG, auditory, premotor and supplementary motor cortex, suggesting that orchestrated processing in the whole dorsal auditory stream is necessary for the complex auditory–motor translation and control processes needed to shadow lifelike AV speech. We also obtained results indicating that auditory–motor translation during speech shadowing may be mediated through a circuitry involving the posterior auditory and premotor cortices, as faster shadowing responses modulated responsivity in these regions.

## Supplementary Information

Supplementary Figure 1.

## Data Availability

Due to concerns regarding participant privacy, structural MRI data and raw functional MRI data will not be made openly available. However, anonymized fMRI data which have been transformed into standard space and behavioural data may be made openly available. The data used to generate the figures in the present study are shared using the Open science framework under Attention and Memory Networks (https://osf.io/agxth/). Other anonymized data is available from the corresponding author on reasonable request. The computer code used to derive the findings of the present study is available from the corresponding author upon reasonable request.

## References

[1] Tremblay P, Dick AS (2016). Broca and Wernicke are dead, or moving past the classic model of language neurobiology. Brain Lang..

[2] Liberman AM, Harris KS, Hoffman HS, Griffith BC (1957). The discrimination of speech sounds within and across phoneme boundaries. J. Exp. Psychol..

[3] Hickok G (2012). Computational neuroanatomy of speech production. Nat. Rev. Neurosci..

[4] Buchsbaum BR, Hickok G, Humphries C (2001). Role of left posterior superior temporal gyrus in phonological processing for speech perception and production. Cogn. Sci..

[5] Peschke C, Ziegler W, Kappes J, Baumgaertner A (2009). Auditory–motor integration during fast repetition: The neuronal correlates of shadowing. Neuroimage.

[6] Burnett TA, Freedland MB, Larson CR, Hain TC (1998). Voice F0 responses to manipulations in pitch feedback. J. Acoust. Soc. Am..

[7] Purcell DW, Munhall KG (2006). Compensation following real-time manipulation of formants in isolated vowels. J. Acoust. Soc. Am..

[8] Tourville JA, Reilly KJ, Guenther FH (2008). Neural mechanisms underlying auditory feedback control of speech. Neuroimage.

[9] Rauschecker JP, Scott SK (2009). Maps and streams in the auditory cortex: Nonhuman primates illuminate human speech processing. Nat. Neurosci..

[10] Rauschecker JP (2010). An expanded role for the dorsal auditory pathway in sensorimotor control and integration. Hear. Res..

[11] Rauschecker JP, Frühholz S, Belin P, Rauschecker JP (2018). Dual stream models of auditory vocal communication. The Oxford Handbook of Voice Perception.

[12] DeWitt I, Rauschecker JP (2012). Phoneme and word recognition in the auditory ventral stream. Proc. Natl. Acad. Sci..

[13] Hickok G, Poeppel D (2007). The cortical organization of speech processing. Nat. Rev. Neurosci..

[14] Hickok G, Ulmer S, Jansen O (2010). The functional anatomy of speech processing: From auditory cortex to speech recognition and speech production. fMRI.

[15] Hickok G, Okada K, Serences JT (2009). Area spt in the human planum temporale supports sensory-motor integration for speech processing. J. Neurophysiol..

[16] Hickok G, Buchsbaum B, Humphries C, Muftuler T (2003). Auditory–motor interaction revealed by fMRI: Speech, music, and working memory in area Spt. J. Cogn. Neurosci..

[17] Pa J, Hickok G (2008). A parietal-temporal sensory-motor integration area for the human vocal tract: Evidence from an fMRI study of skilled musicians. Neuropsychologia.

[18] Hickok G (2016). A cortical circuit for voluntary laryngeal control: Implications for the evolution language. Psychon. Bull. Rev..

[19] Baldo JV, Klostermann EC, Dronkers NF (2008). It's either a cook or a baker: Patients with conduction aphasia get the gist but lose the trace. Brain Lang..

[20] Buchsbaum BR (2011). Conduction aphasia, sensory-motor integration, and phonological short-term memory—An aggregate analysis of lesion and fMRI data. Brain Lang..

[21] Parker Jones OP (2014). Sensory-to-motor integration during auditory repetition: A combined fMRI and lesion study. Front. Hum. Neurosci..

[22] Rogalsky C (2015). Speech repetition as a window on the neurobiology of auditory–motor integration for speech: A voxel-based lesion symptom mapping study. Neuropsychologia.

[23] Simmonds AJ (2014). Parallel systems in the control of speech. Hum. Brain Mapp..

[24] Simmonds AJ, Leech R, Collins C, Redjep O, Wise RJS (2014). Sensory-motor integration during speech production localizes to both left and right plana temporale. J. Neurosci..

[25] Wikman P, Rinne T (2018). Interaction of the effects associated with auditory–motor integration and attention-engaging listening tasks. Neuropsychologia..

[26] Wilson SM, Iacoboni M (2006). Neural responses to non-native phonemes varying in producibility: Evidence for the sensorimotor nature of speech perception. Neuroimage.

[27] Wikman P (2020). Breaking down the cocktail party: Attentional modulation of cerebral audiovisual speech processing. NeuroImage.

[28] Richter D, Ekman M, de Lange FP (2018). suppressed sensory response to predictable object stimuli throughout the ventral visual stream. J. Neurosci..

[29] Porter R, Lubker J (1980). Rapid reproduction of vowel-vowel sequences: Evidence for a fast and direct acoustic-motoric linkage in speech. J. Speech Lang. Hear. Res..

[30] Cherry EC (1953). Some experiments on the recognition of speech, with one and with two ears. J. Acoust. Soc. Am..

[31] Alho K (2003). Hemispheric lateralization of cerebral blood-flow changes during selective listening to dichotically presented continuous speech. Brain Res. Cogn. Brain Res..

[32] Alho K (2006). Selective attention to human voice enhances brain activity bilaterally in the superior temporal sulcus. Brain Res..

[33] Leminen A (2020). Modulation of brain activity by selective attention to audiovisual dialogues. Front. Neurosci..

[34] McGettigan C (2012). Speech comprehension aided by multiple modalities: Behavioural and neural interactions. Neuropsychologia.

[35] Marinato G, Baldauf D (2019). Object-based attention in complex, naturalistic auditory streams. Sci. Rep..

[36] de Vries IE, Marinato G, Baldauf D (2021). Decoding object-based auditory attention from source-reconstructed MEG alpha oscillations. J. Neurosci..

[37] Ylinen A, Wikman P, Leminen M, Alho K (2022). Task-dependent cortical activations during selective attention to audiovisual speech. Brain Res..

[38] Binder JR, Desai RH, Graves WW, Conant LL (2009). Where is the semantic system? A critical review and meta-analysis of 120 functional neuroimaging studies. Cereb. Cortex.

[39] Mottonen R, Watkins KE (2009). Motor representations of articulators contribute to categorical perception of speech sounds. J. Neurosci..

[40] Davis MH, Johnsrude IS (2003). Hierarchical processing in spoken language comprehension. J. Neurosci..

[41] Sumby WH, Pollack I (1954). Visual contribution to speech intelligibility in noise. J. Acoust. Soc. Am..

[42] Shuster LI, Lemieux SK (2005). An fMRI investigation of covertly and overtly produced mono- and multisyllabic words. Brain Lang..

[43] Oldfield RC (1971). The assessment and analysis of handedness: The Edinburgh inventory. Neuropsychologia.

[44] Shannon RV, Zeng F-G, Kamath V, Wygonski J, Ekelid M (1995). Speech recognition with primarily temporal cues. Science.

[45] Boersma, P. & Weenink, D. *Praat Speech Processing Software*. http://www.praat.org (Institute of Phonetics Sciences of the University of Amsterdam, 2001). Retrieved June 5, 2018.

[46] Posner MI, Cohen Y (1984). Components of visual orienting. Attent. Perform. X Control Lang. Process..

[47] Birn RM, Cox RW, Bandettini PA (2004). Experimental designs and processing strategies for fMRI studies involving overt verbal responses. Neuroimage.

[48] Mullinger K, Debener S, Coxon R, Bowtell R (2008). Effects of simultaneous EEG recording on MRI data quality at 1.5, 3 and 7 tesla. Int. J. Psychophysiol..

[49] Twilhaar JN, van den Bogaerde B (2016). Concise Lexicon for Sign Linguistics.

[50] Jenkinson M, Smith S (2001). A global optimisation method for robust affine registration of brain images. Med. Image Anal..

[51] Jenkinson M, Bannister P, Brady M, Smith S (2002). Improved optimization for the robust and accurate linear registration and motion correction of brain images. Neuroimage.

[52] Smith SM (2002). Fast robust automated brain extraction. Hum. Brain Mapp..

[53] Fischl B (2012). FreeSurfer. Neuroimage.

[54] Greve DN, Fischl B (2009). Accurate and robust brain image alignment using boundary-based registration. Neuroimage.

[55] Wikman PA, Vainio L, Rinne T (2015). The effect of precision and power grips on activations in human auditory cortex. Front. Neurosci..

[56] Adolphs R (2009). The social brain: Neural basis of social knowledge. Annu. Rev. Psychol..

[57] Alcalá-López D, Vogeley K, Binkofski F, Bzdok D (2019). Building blocks of social cognition: Mirror, mentalize, share?. Cortex.

[58] Bzdok D, Laird AR, Zilles K, Fox PT, Eickhoff SB (2013). An investigation of the structural, connectional, and functional subspecialization in the human amygdala. Hum. Brain Mapp..

[59] Mitchell JP (2006). Mentalizing and Marr: An information processing approach to the study of social cognition. Brain Res..

[60] Leminen A (2020). Modulation of brain activity by selective attention to audiovisual dialogues. Front. Neurosci..

[61] Romanski LM (2012). Integration of faces and vocalizations in ventral prefrontal cortex: Implications for the evolution of audiovisual speech. Proc. Natl. Acad. Sci..

[62] Häkkinen S, Rinne T (2018). Intrinsic, stimulus-driven and task-dependent connectivity in human auditory cortex. Brain Struct. Funct..

[63] Rinne T, Koistinen S, Salonen O, Alho K (2009). Task-dependent activations of human auditory cortex during pitch discrimination and pitch memory tasks. J. Neurosci..

[64] Rinne T, Koistinen S, Talja S, Wikman P, Salonen O (2011). Task-dependent activations of human auditory cortex during spatial discrimination and spatial memory tasks. Neuroimage.

[65] Ahveninen J (2006). Task-modulated “what” and “where” pathways in human auditory cortex. Proc. Natl. Acad. Sci..

[66] Alho K, Rinne T, Herron TJ, Woods DL (2014). Stimulus-dependent activations and attention-related modulations in the auditory cortex: A meta-analysis of fMRI studies. Hear. Res..

[67] Häkkinen S, Ovaska N, Rinne T (2015). Processing of pitch and location in human auditory cortex during visual and auditory tasks. Front. Psychol..

[68] DeWitt I, Rauschecker JP (2012). Phoneme and word recognition in the auditory ventral stream. Proc. Natl. Acad. Sci..

[69] Petkov CI (2004). Attentional modulation of human auditory cortex. Nat. Neurosci..

[70] Rauschecker JP, Romanski LM, Winer JA, Schreiner CE (2011). Auditory cortical organization: Evidence for functional streams. The Auditory Cortex.

[71] Friederici AD, Alter K (2004). Lateralization of auditory language functions: A dynamic dual pathway model. Brain Lang..

[72] Agnew ZK, McGettigan C, Banks B, Scott SK (2013). Articulatory movements modulate auditory responses to speech. Neuroimage.

[73] Eliades SJ, Wang XQ (2003). Sensory-motor interaction in the primate auditory cortex during self-initiated vocalizations. J. Neurophysiol..

[74] Greenlee JDW (2013). Sensory-motor interactions for vocal pitch monitoring in non-primary human auditory cortex. PLoS ONE..

[75] Schroeger E, Marzecova A, SanMiguel I (2015). Attention and prediction in human audition: A lesson from cognitive psychophysiology. Eur. J. Neurosci..

[76] Guenther FH, Vladusich T (2012). A neural theory of speech acquisition and production. J. Neurolinguist..

[77] Peschke C, Ziegler W, Eisenberger J, Baumgaertner A (2012). Phonological manipulation between speech perception and production activates a parieto-frontal circuit. Neuroimage..

[78] Price CJ (2010). The anatomy of language: A review of 100 fMRI studies published in 2009. Ann. N. Y. Acad. Sci..

[79] Tremblay S, Shiller DM, Ostry DJ (2003). Somatosensory basis of speech production. Nature.

[80] Hickok G (2014). Partially overlapping sensorimotor networks underlie speech praxis and verbal short-term memory: Evidence from apraxia of speech following acute stroke. Front. Hum. Neurosci..

[81] Chein JM, Schneider W (2012). The brain's learning and control architecture. Curr. Dir. Psychol. Sci..

[82] Simmonds AJ, Wise RJS, Dhanjal NS, Leech R (2011). A comparison of sensory-motor activity during speech in first and second languages. J. Neurophysiol..

