# Supplementary material for: Brain activity during shadowing of audiovisual cocktail party speech, contributions of auditory–motor integration and selective attention
Source: Sci Rep. 2022 Nov 5;12:18789. doi: 10.1038/s41598-022-22041-2 (PMC9637225; doi:10.1038/s41598-022-22041-2)
Supplement: Supplementary file 1 — Supplementary Figure 1. [file 41598_2022_22041_MOESM1_ESM.docx]

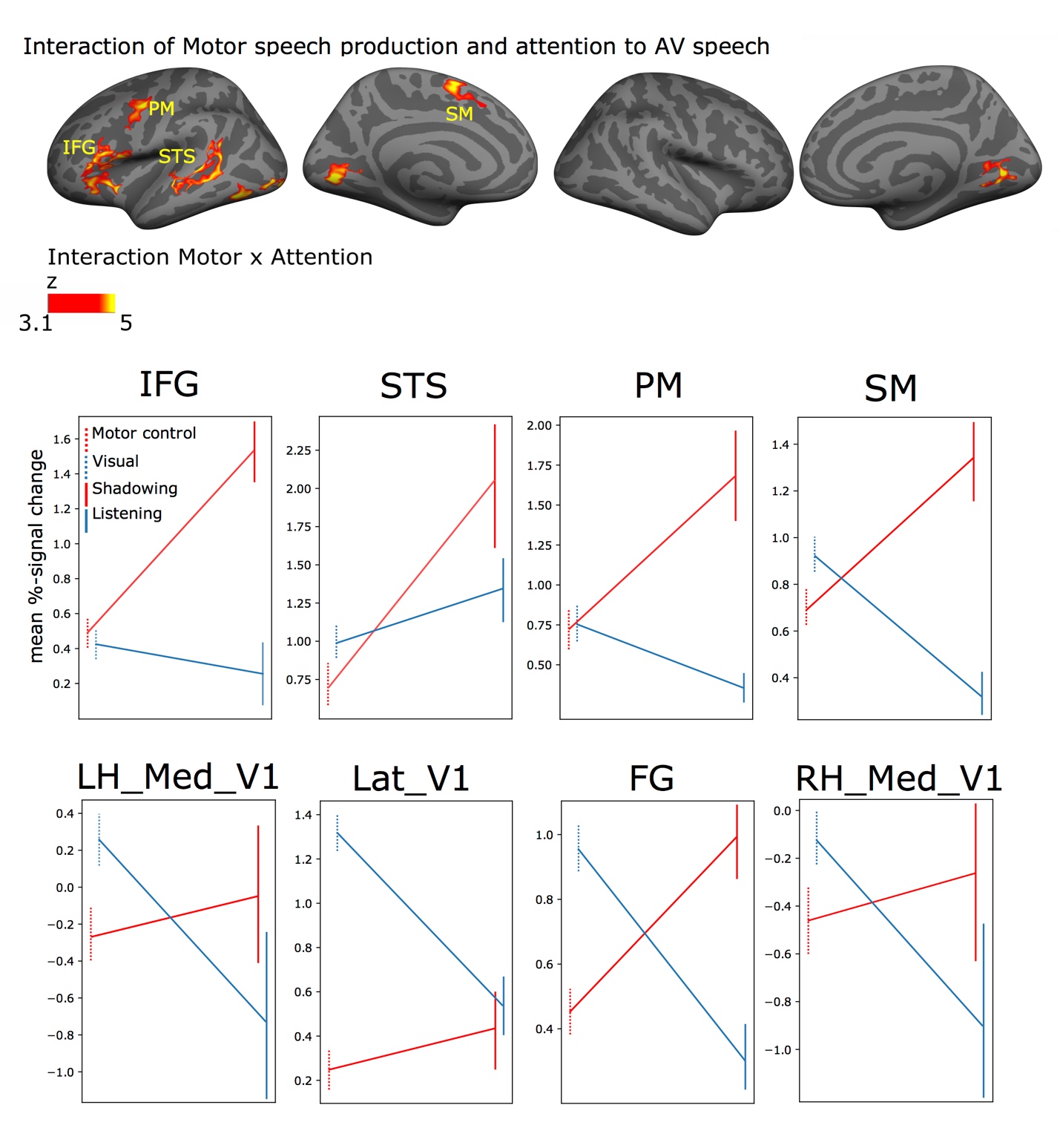


**Supplementary Figure1. Significant interactions between Motor speech production and Attention to AV speech were found in left hemisphere auditory, motor and language regions. Top row:**  Significant clusters (initial cluster threshold z = 3.1; permutated cluster significance p < 0.05, FWER corrected) for the interaction Motor speech production × Attention to AV speech. From left to right: lateral and medial views of the inflated left hemisphere and lateral and medial views of the right hemisphere (lighter gray denotes gyri and darker gray sulci). **Bottom row:** The mean % signal change (vs. baseline; y-axis) in each of the tasks are plotted separately for all significant clusters. Note that interaction effects in the V1 arose because the attention effect was stronger when comparing the listening task to the visual control task than when comparing the motor control task to the shadowing task, which was somewhat expected, since the motor control task did not explicitly divert attention from the faces in the dialogue videos. Interaction effects in the FG and med_V1 seem to arise from these regions specifically being activated during the visual control task, possibly due to processing of the cross and + stimuli (similar findings have been reported in Wikman et al., 2020 and Ylinen et al., 2021 who used the same visual control task). Error bars represent ±SEM. Abbrevations: IFG: inferior frontal gyrus; STS: superior temporal gyrus; PM: premotor cortex; SM: Supplementary motor cortex; Lat_V1: lateral visual are V1; FG: fusiform gyrus; med_V1: medial V1; LH: left hemisphere; RH: right hemisphere.
